# Average volume reference space for large scale registration of whole-body magnetic resonance images

**DOI:** 10.1371/journal.pone.0222700

**Published:** 2019-10-01

**Authors:** Martino Pilia, Joel Kullberg, Håkan Ahlström, Filip Malmberg, Simon Ekström, Robin Strand

**Affiliations:** 1 Department of Surgical Sciences, Uppsala University, Uppsala, Sweden; 2 Department of Information Technology, Uppsala University, Uppsala, Sweden; 3 Antaros Medical, Uppsala, Sweden; Linköping University, SWEDEN

## Abstract

**Background and objectives:**

The construction of whole-body magnetic resonance (MR) imaging atlases allows to perform statistical analysis with applications in anomaly detection, longitudinal, and correlation studies. Atlas-based methods require a common coordinate system to which all the subjects are mapped through image registration. Optimisation of the reference space is an important aspect that affects the subsequent analysis of the registered data, and having a reference space that is neutral with respect to local tissue volume is valuable in correlation studies. The purpose of this work is to generate a reference space for whole-body imaging that has zero voxel-wise average volume change when mapped to a cohort.

**Methods:**

This work proposes an approach to register multiple whole-body images to a common template using volume changes to generate a synthetic reference space, starting with an initial reference and refining it by warping it with a deformation that brings the voxel-wise average volume change associated to the mappings of all the images in the cohort to zero.

**Results:**

Experiments on fat/water separated whole-body MR images show how the method effectively generates a reference space neutral with respect to volume changes, without reducing the quality of the registration nor introducing artefacts in the anatomy, while providing better alignment when compared to an implicit reference groupwise approach.

**Conclusions:**

The proposed method allows to quickly generate a reference space neutral with respect to local volume changes, that retains the registration quality of a sharp template, and that can be used for statistical analysis of voxel-wise correlations in large datasets of whole-body image data.

## Introduction

Whole-body volume image acquisition techniques such as magnetic resonance imaging (MRI), computed tomography (CT), and positron emission tomography (PET) allow to acquire images of anatomy and metabolic processes through the whole organism within minutes. Such imaging methods evolved significantly in the recent past, reaching wide-spread adoption in medical applications, and several institutions are creating massive datasets of whole-body images, such as UK-BioBank (http://www.ukbiobank.ac.uk, with a target of 100 000 subjects) and German Cohort Biobank (http://www.nationale-kohorte.de, 30 000 subjects). Great amounts of high-resolution data open new and unprecedented possibilities for medical research, allowing statistical studies on wide samples that take into account the local composition of the body in all its regions. This has special interest in the study of systemic diseases, such as cancer or metabolic syndrome, whose effects span through the whole body.

The full exploitation of such amount of data prompts to develop methodologies that can take advantage of all the information in the volume, beyond classical approaches based on measurements on regions of interest (ROI), which reduce the analysis to a handful of selected quantities. The possibility to build an MRI whole-body atlas has been demonstrated in previous studies, showing the effectiveness of whole-body image-driven statistical analysis, a methodology presented as Imiomics (imaging-omics), with applications to anomaly detection for automatic recognition of systemic diseases such as cancer through all the body, longitudinal studies to measure the effectiveness of treatments against these diseases, and correlation studies to explore relationships between the local composition of the body and non-image biomarkers, and the correlation of both with health or pathology conditions [1–3]. In such studies, pointwise tissue volume, estimated through the Jacobian of the deformation that maps the reference space to a subject, is a quantity of interest for correlation studies. In order to prevent a bias due to a non-normalised reference space, it is of interest to find a reference that is neutral with respect to pointwise tissue volume, meaning that the average volume change when mapping a point from the reference to the whole cohort is zero.

The construction of an anatomical atlas requires point-to-point correspondences, often obtained by determining a common reference coordinate space to which all the images are mapped in the registration process. In order to maximise the quality of image registration and minimise the bias due to the choice of the reference space, an optimisation process of the reference space is necessary. Early approaches used a template image selected from the cohort as the reference space, to which all the other images were then registered independently; the inconvenience of this methodology is the need to explicitly select the reference space from the dataset, since it is unlikely to find a template that fits all the subjects under study, due to local and global anatomical variations, and it imposes an unavoidable bias toward the features of the selected subject.

Groupwise image registration was introduced in the context of neuroimaging to solve these issues. It consists of a registration process that defines a collection of transforms toward all the images to be registered, which are optimised simultaneously targeting a cost measure defined over the whole dataset, producing a reference space and a set of mappings from such space that are optimal for the cohort as a whole. Significant effort has been devoted to research on groupwise methods, leading to the development of a large collection of techniques that rely on different principles. Most existing groupwise approaches create an implicit template whose synthesis happens within the optimisation process itself [4, 5]. One drawback of many implicit reference methods is that they do not generate a sharp template, and a mean image must be recovered from the deformed moving images.

While implicit-reference groupwise registration methods are not biased toward a choice of the reference and have strong theoretical properties, independent pairwise registration of the subjects to an explicit reference has some practical advantages, especially when dealing with large datasets. It requires significantly less resources, in terms of computing time and memory footprint, while groupwise registration can become unfeasible when the size of the images or the number of registered subjects is large. Another practical advantage in the processing of big datasets is that, when performing pairwise registration, it is straightforward to distribute calculations among different machines.

This work proposes an approach to optimise a reference space for the registration of multiple whole-body images, producing a synthetic reference having zero average volume change in each voxel when mapped to the whole cohort. A suitable initial reference is selected from the images within the cohort and all subjects are independently registered to it with a process referred as pairwise registration. A map of the average pointwise volume change from the mapping of the reference to the subjects cohort is generated, and used to produce a deformation whose point-wise volume change matches the aforementioned map, which in turn is used to resample a new reference.

The method is conceptually similar to previous approaches proposed in the field of neuroimaging to generate a synthetic reference by deforming an initial image with a transform computed from the average of a residual deformation field [6, 7]. In a whole-body registration setting the assumptions behind these methods may not hold. For instance, even assuming consistent pose of the subjects in the acquisition process, it is generally not easy to decompose the deformation between an affine component, accounting for global alignment and scale, and an elastic residual, accounting for purely morphological differences in the anatomy of the subjects. For this reason, instead of relying directly on residual and average deformation fields to update the reference, the usage of an adequately defined average volume change to guide the refinement of the reference space is explored.

The approach was tested on a dataset of whole-body MR images, showing how the proposed method generates a synthetic reference with zero average voxel-wise volume changes, without introducing artefacts in the anatomy nor negatively affecting the quality of the registration.

To summarise, the original contribution of this work is

- the introduction of an average volume reference space for voxel-wise statistical analysis of large datasets of whole-body image data;
- the formulation of a method for its fast generation;
- an open-source implementation of the required software tools.

## Theory

The transform that registers a moving image $I_{j}:\Omega_{j}\to R$ to a fixed image $I_{R}:\Omega_{R}\to R$ can be expressed as a deformation field *f*_*j*_(***x***): Ω_*R*_ → Ω_*j*_ that associates to each point ***x*** = (*x*_1_, …, *x*_*n*_)∈Ω_*R*_ in *I*_*R*_ the coordinates ***x***′ of its corresponding position in *I*_*j*_. This deformation is used in practice to resample the warped image by taking samples in the moving image space from coordinates in the reference space without holes. The deformation can be decomposed as the sum of the identity function id and a displacement *u*

$$\begin{array}{c} f(x)=\mathrm{id}(x)+u(x). \end{array}$$

The Jacobian determinant *J*[*f*](***x***) associated to a function $f\left(x\right)=\left(f_{1}\left(x\right),\ldots ,f_{n}\left(x\right)\right):\Omega \subseteq R^{n}\to R^{n}$ is defined as the determinant of the Jacobian matrix

$$\begin{array}{c} J\left[f\right]\left(x\right)=\det{\left(\frac{\partial f_{i}\left(x\right)}{\partial x_{j}}\right)}_{ij}. \end{array}$$

The physical interpretation of the Jacobian determinant of the transform produced by an image registration process is the quantification of the local volume change associated to it. If the value of the Jacobian at a certain location ***x*** is unitary then the function does not cause a local volume change in ***x***, a value above 1 implies a local expansion of the space, a value between 0 and 1 implies a local contraction. A Jacobian of 0 implies a local degeneration of the space to a null set, while values below 0 denote a function that is locally folding the space, inverting its orientation. The last two cases represent a non-invertible and physically unfeasible transform.

**Fig 1:**
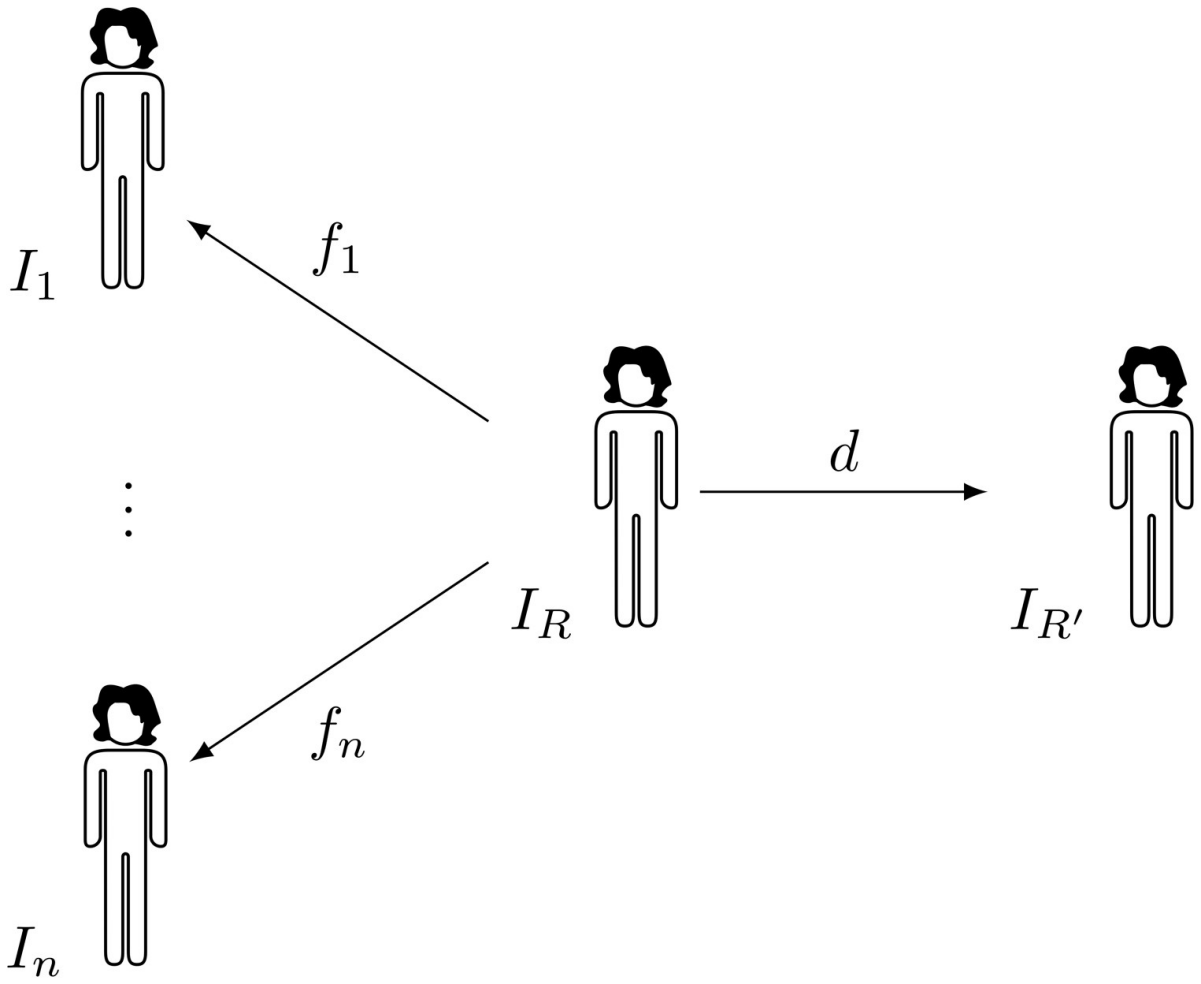
Diagram showing the transformations between images. Given a cohort of *n* subjects *I*_1_, …, *I*_*n*_ registered to the initial reference image *I*_*R*_ by the deformations *f*_1_, …, *f*_*n*_, a new reference image *I*_*R*′_ is obtained warping *I*_*R*_ by a deformation *d* whose Jacobian is the geometric mean of the Jacobians of *f*_1_, …, *f*_*n*_.

Given a set of *n* images $I=\{I_{1},\ldots ,I_{n}\}$ and an initial reference image $I_{R}\in I$, a new implicit reference $I_{R^{\prime }}:\Omega_{R^{\prime }}\to R$ is defined, related to *I*_*R*_ through a deformation field *d*: Ω_*R*_ → Ω_*R*′_. The deformation that registers *I*_*j*_ to *I*_*R*′_ is given by *f*_*j*_ ○ *d*^−1^: Ω_*R*′_ → Ω_*j*_ (Fig 1). The voxel-wise product of the Jacobian determinants *J* of these deformations is given by

$$\begin{array}{c} \prod_{i=1}^{n}J[f_{i}○d^{-1}]=\prod_{i=1}^{n}J[f_{i}]\cdot J[d^{-1}]=\frac{\prod_{i=1}^{n}J[f_{i}]}{{\left(J[d]\right)}^{n}} \end{array}$$

and imposing it to be equal to 1, equivalent to no average pointwise volume change, leads to

$$\begin{array}{c} J[d]=\sqrt[{n}]{\prod_{i=1}^{n}J[f_{i}]}. \end{array}$$ (1)

Determining the transform *d* is non-trivial in practice since, without boundary conditions, the generation of a deformation with prescribed Jacobian determinant is an ill-posed problem. Different approaches were developed in neuroimaging, where this problem is of interest for the generation of synthetic ground truth for the evaluation of algorithms for automated measure of brain atrophy. Van Eede et al. [8] and Karaçali et al. [9] propose two different search-based methods. Khanal et al. [10] propose a biophysical model for tissue atrophy, whose associated system of equations is solved numerically with the finite difference method. Other authors [11, 12] formulate the atrophy as an elastic deformation problem and solve it numerically with the finite element method.

While the nature of the problem is the same, in this context additional difficulties arise, due to the different properties of the average Jacobian map. While synthetic atrophy maps used to generate synthetic deformations in neuroimaging studies are usually piecewise constant functions with relatively small magnitude, the average Jacobian in this work is a continuous function with a complicated landscape, that attains very high or low values in some regions.

## Materials and methods

The proposed method takes as input a set of images, and generates a synthetic reference and a set of deformations that map the reference space to all the input images (Fig 1). This is achieved in two stages, first registering all images to an initial reference selected from the dataset, and then determining the pointwise average volume change from the initial reference to the rest of the cohort and generating a new reference that compensates them.

### Image data

The proposed method is evaluated on whole-body fat/water separated MR images, in order to observe its effect on the quality of the registration results.

The experiments are conducted on a dataset of 167 female and 159 male subjects from the Prospective investigation of Obesity, Energy and Metabolism (POEM, http://www.medsci.uu.se/poem), scanned in Uppsala, Sweden, in the context of an investigation of links between obesity and cardiovascular disorders, composed by a sample of 50-year old inhabitants of the city. Ethical approval for the study was obtained from the Regional Ethical Review Board in Uppsala, Sweden (Approval numbers: Uppsala Dnr 2009/057 and Dnr 2012/143), and written consent was obtained from all subjects.

In this work, the image data for each subject is composed by a couple of whole body images with absolute fat and water content, which are inherently co-registered from the acquisition. The images were corrected for intensity inhomogeneity by slice-wise normalisation of intensity values and simultaneous correction (SIM) [13]. In addition, for each subject a binary mask that segments the body from the background was computed, obtained by fuzzy c-means clustering followed by morphological refinement. The field of view is 530 × 377 × 2000 mm^3^ (left-right × front-back × foot-head), sampled in an orthogonal grid of resolution 256 × 256 × 252, with voxel size 2.07 × 2.07 × 8.00 mm^3^. In addition to the image data, the dataset includes measures of several non-image biomarkers. Details regarding acquisition and preprocessing are described by Strand et al. [1].

### Selection of the initial reference

The initial reference image is defined by selecting an image from the cohort. The criterion used is to select a subject that is closest to both the median fat mass and median height of the cohort, excluding images with evident problems such as artefacts, anatomical anomalies, or parts of the body outside the field of view. Observation suggests that median fat mass gives a reference with a low average volume change, since fat deposits host most of the variability.

Image resampling introduces a small amount of smoothing that affects the distribution of intensities and has a measurable impact on the image similarity metrics. In order to compensate for this effect when comparing results registered on the initial reference and on a synthetic one, the initial registration is not performed on the image of the initial reference from the dataset, but on a resampled image obtained applying a translation of half voxel along all axes, in order to introduce the smoothing effect of resampling without altering the anatomy.

### Image registration

In principle, image registration can be performed using any pairwise method that is suitable for the dataset. In this work, a fast iterative method based on graph cuts [14], is used. The energy function is in the form

$$\begin{array}{c} E\left(f\right)=\sum_{I\in I}w_{I}\sum_{p\in \Omega }{\left|I_{R}\left(p\right)-I\left(p+u\left(p\right)\right)\right|}^{2}+\sum_{(p,q)\in N}{∥u(p)-u(q)∥}^{2} \end{array}$$

using as data term the sum of squared difference of image intensities over the image channels I∈I and as smoothness term a diffusion regulariser [15] that penalises high first order derivatives, with N denoting the set of 6-connected neighbouring voxel pairs in the image.

### Generation of deformations from known volume changes

The average Jacobian, as defined in Eq 1, is computed numerically with the following formula

$$\begin{array}{c} \bar{J}=e^{\frac{1}{n}\sum_{i=1}^{n}\ln(\min\{\epsilon ,J_{i}\})} \end{array}$$

where *J*_*i*_ is the Jacobian of the deformation field from the registration of the *i*-th subjects to the reference. The average is computed under logarithm for numerical stability, and a lower threshold *ϵ* > 0 is imposed to remove singularities due to local folds of the images that can derive from errors in the registration process.

The displacement is generated from the Jacobian using a search-based approach similar to the one proposed by van Eede et al. [8].

Other approaches such as the one proposed by Khanal et al. [10] were explored and excluded due to excessive demand of computational resources when applied to images of the size used in this work, while FEM-based methods [11, 12] were excluded to avoid the non-trivial problem of generating anatomically meaningful volume meshes with good numerical properties for a volume as complex as the whole human body.

The search-based method used in this work was here reimplemented in a high-performance version with GPU acceleration, and made freely available as open-source software, together with a wrapping Python package (https://github.com/m-pilia/disptools).

### Evaluation metrics

Ground truth segmentation of several organs is available for a subset of the dataset, and the comparative evaluation is based on the Sørensen–Dice coefficient [16] between the labelling in reference space and the warped segmentation of the moving image. Since no segmentation exists for the synthetic reference spaces, a majority voting procedure is used to determine the label assignment [7].

Other estimators computed include average inverse consistency error (AICE) [17] of the transform *h* obtained from the registration

$$AICE(h,h_{I})=\frac{1}{\left|\Omega \right|}\int_{\Omega }∥\left(h_{I}○h\right)\left(x\right)-x∥\text{d}x$$

where *h*_*I*_ is the transform obtained when inverting the roles of fixed and moving image, mean squared error (MSE)

$$MSE(I_{R},I_{J})=\frac{1}{\left|\Omega \right|}\int_{\Omega }{\left(I_{R}\left(x\right)-I_{j}\left(h\left(x\right)\right)\right)}^{2}\text{d}x$$

and mutual information (MI)

$$MI(I_{R},I_{J})={\;\int \int \;}_{x\in r_{R},y\in r_{j}}p_{R,I_{j}}\left(x,y\right)\log(\frac{p_{R,I_{j}}\left(x,y\right)}{p_{R}\left(x\right)p_{I_{j}}\left(y\right)})\text{d}x\text{d}y$$

between the warped subject *I*_*j*_ and the reference image *I*_*R*_ for each registration, within a region of interest (ROI) Ω denoting the body volume. The intensity range of the reference and moving image are denoted as *r*_*R*_ and *r*_*j*_ respectively. The marginal and joint probability density distributions $p_{R,I_{j}},p_{R},p_{I_{j}}$ involved in the computation of mutual information were approximated with 256 bin histograms of the image within the ROI.

It must be noted that inverse consistency and intensity-based measures do not represent a reliable estimation of registration quality [18], but they provide a generic estimation of matching whereas ground truth segmentation is not available for most of the dataset.

Since the Jacobian is not a linear function of the volume change, it is not meaningful to directly compute squared differences of the Jacobian. For this reason, an additional transform

$$\begin{array}{c} V\left[h\right]\left(x\right)=\{\begin{array}{cc} 1-\frac{1}{J[h](x)}\; & J[h](x)\in (0,1)\\ J[h](x)-1\; & J[h](x)\geq 1 \end{array} \end{array}$$ (2)

is defined for convenience to express volume changes giving the same importance to compression and expansion. The average absolute volume change (AAVC) for a subject is defined as

$$\begin{array}{c} AAVC\left(h\right)=\frac{1}{\left|\Omega \right|}\int_{\Omega }|V[h](x)|\text{d}x \end{array}$$ (3)

where *h* is the transform that registers the subject to the reference.

### Experiment overview

Three different experiments were conducted:

1. A first experiment to demonstrate the generation of a synthetic reference in the registration of two different sets of images, containing respectively all the female and male subjects.
2. A second experiment to show the stable behaviour of the method when iteratively refining the synthetic reference with iterated registration processes, using a subset of 70 female subjects.
3. A third experiment to compare the outcome of the method with a well established implicit-reference groupwise registration approach, using a subset of 25 female subjects.

Image registration was performed on a desktop workstation equipped with an AMD Ryzen 7 2700 X CPU @ 3.70 GHz, taking approximately 1-2 minutes per subject. The displacement used to resample the synthetic reference from the initial one was obtained from the average Jacobian determinant, running our CUDA implementation of the algorithm on a Nvidia GTX 1080Ti GPU, with the computation requiring approximately 2-3 minutes per reference.

All the deformations were encoded as dense displacement fields, and the displacements that register the subjects to the initial reference were composed with the inverse of the displacement that generates the synthetic reference to generate the final displacement (Fig 1). Image resampling and displacement field inversion were computed using filters from the Insight Toolkit (ITK) [19]. These operations introduce a certain amount of error, whose impact was estimated by refining the registration of the subjects on the synthetic reference, using the composed displacement as initial deformation for a new registration process. The registration method used in this experiment can take advantage of such initialisation, that approximately halves the computing time.

### Experiment on registration of a large dataset

A first experiment demonstrates the generation of a synthetic reference for a large scale study on whole-body MR volume images. Male and female subjects were treated as two different cohorts, and for each cohort all subjects were registered to an initial reference closest to the median of both of fat mass and height for the cohort.

After the initial registration, a synthetic reference was generated for each cohort with the proposed method, and the deformations that register each subject to this new reference were obtained by composing the displacement between the references with the displacement that registered each subject to the initial reference.

The effect of refining the deformation on the synthetic reference was also evaluated, by running a second registration using the composed deformation obtained so far as input.

### Experiment on stability evaluation

In order to evaluate the stability of the method, another experiment was performed, using a similar methodology to the previous experiment and, in addition, refining the registration of each subject to the new, synthetic reference, and using the resulting average Jacobian to generate a new reference. This process was iteratively repeated ten times. To prevent degradation of the image quality over the iterations when generating the new reference from the previous, the displacements are composed and the new reference is always sampled directly from the initial reference.

For practical reasons, the data used in this experiment is limited to a subset of 70 subjects from the female cohort, uniformly distributed in terms of fat mass.

### Comparison with implicit-reference groupwise registration

A comparative evaluation with respect to an implicit reference groupwise registration method was performed, using the non-rigid image registration toolbox Elastix [20] version 4.9.0 as the baseline. Elastix provides an open-source implementation of implicit reference groupwise registration based on a B-spline transform model, offering different groupwise image similarity measures, including variance of the intensity [21] and PCA-based metrics [22].

A subset of 25 subjects from the original cohort was used, due to the limitation in terms of available segmentations, and because resource consumption is a limiting factor for Elastix when running implicit reference groupwise registrations over a large set of images.

In the registration with Elastix, subjects were affinely pre-aligned. Groupwise registration was performed with a stacked B-spline transform and with multi-image and multi-resolution settings. In addition to fat and water channels, the binary mask segmenting the body was used as additional channel, to improve robustness. The best results, reported in this work, were achieved using as metric the variance of intensity over the moving images. The complete parameter file used in the experiment is available at https://github.com/m-pilia/WBGroupwiseParameters.

## Results

**Fig 2:**
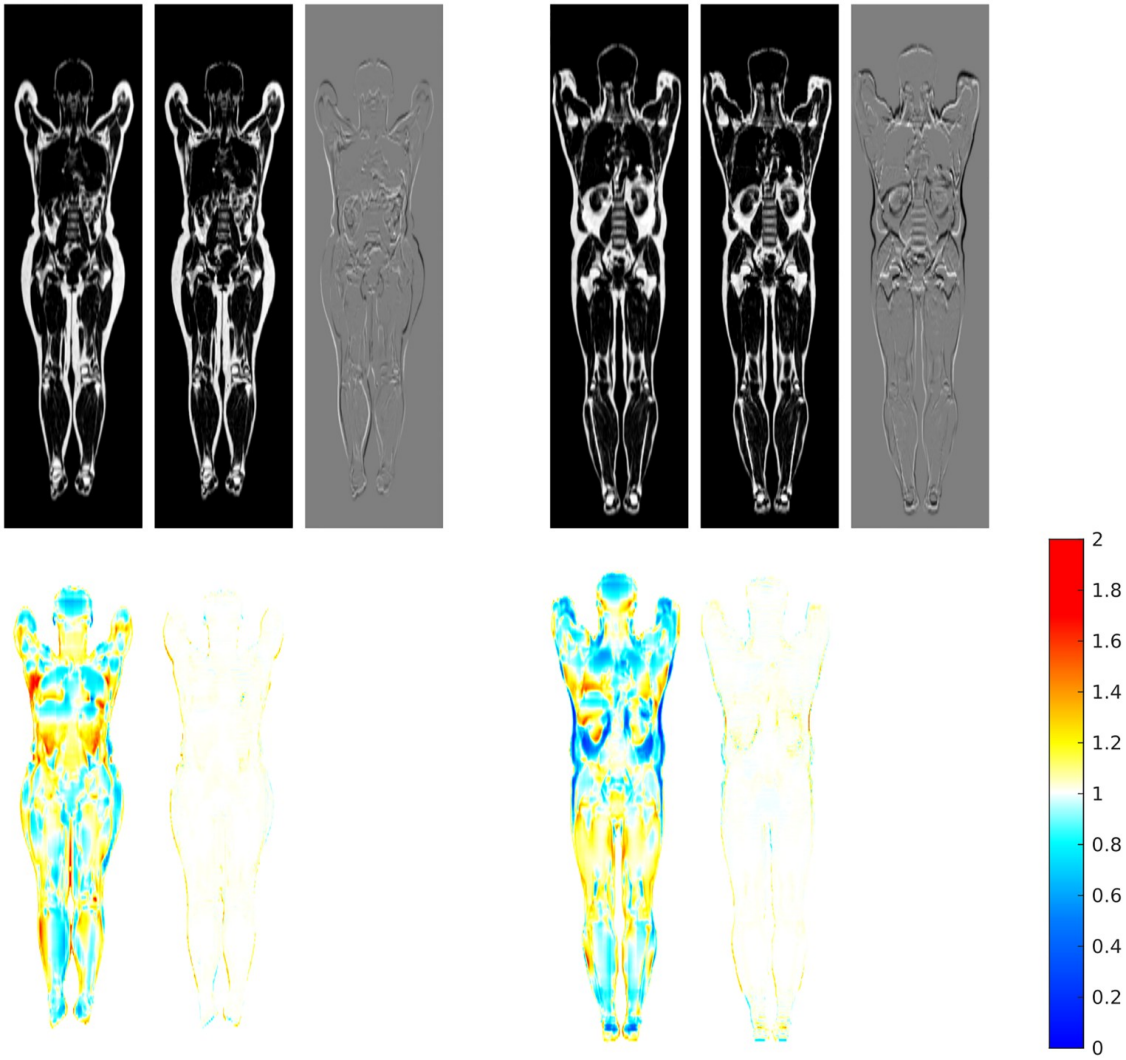
Generation of the synthetic reference for the registration of two different cohorts. Top row: slices of the initial and synthetic reference and difference images for the females (left) and males (right). Bottom row: Jacobian determinant for the initial and synthetic references.

**Fig 3:**
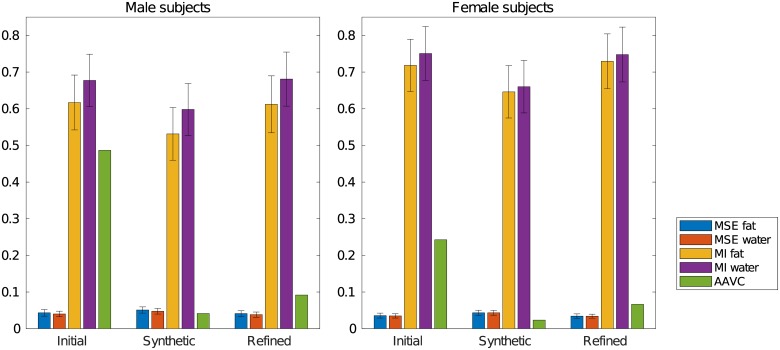
Generation of the synthetic reference for the registration of two different cohorts. Plot of the average absolute volume change (AAVC) as defined in Eq 3, and of the mean and variance across the cohort of mean squared error (MSE) and mutual information (MI) for the registration on the initial reference, the result on the synthetic reference, and a further refined registration on the synthetic reference. The data for each subject is reported in S1 and S2 Files.

Slices of the synthetic reference for the female and male subjects in the dataset, obtained with the proposed method in the first experiment, are reported in Fig 2 together with the Jacobian determinant. Measures collected from the registration on the original and synthetic reference are plotted in Fig 3.

**Fig 4:**
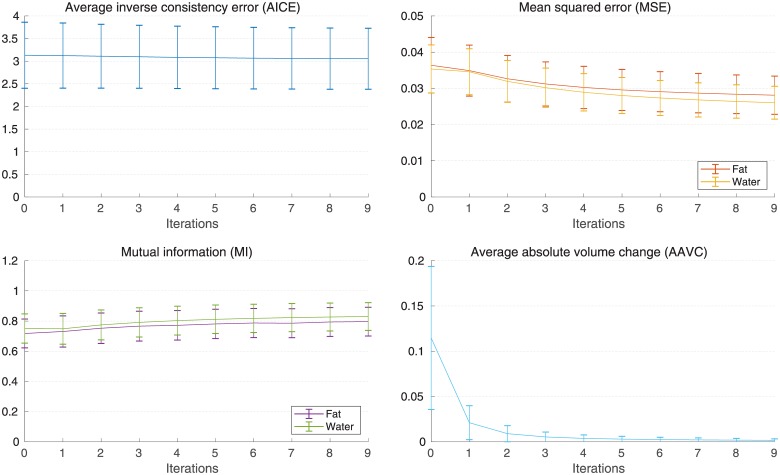
Evaluation of the method stability. Evolution of mean and standard deviation of the measures over the iterations, when iteratively repeating the registration and generation of the synthetic reference. Iteration 0 represents the registration on the initial reference. In the plot of AAVC, to compute the average among the voxels in reference space (Eq 2), outlier voxels whose absolute volume change differs from the median by more than 2.5 times the median absolute deviation were excluded. The data for each subject is reported in S3 File.

The data collected in the second experiment, when repeatedly iterating registration and generation of the synthetic reference, is reported in Fig 4.

**Fig 5:**
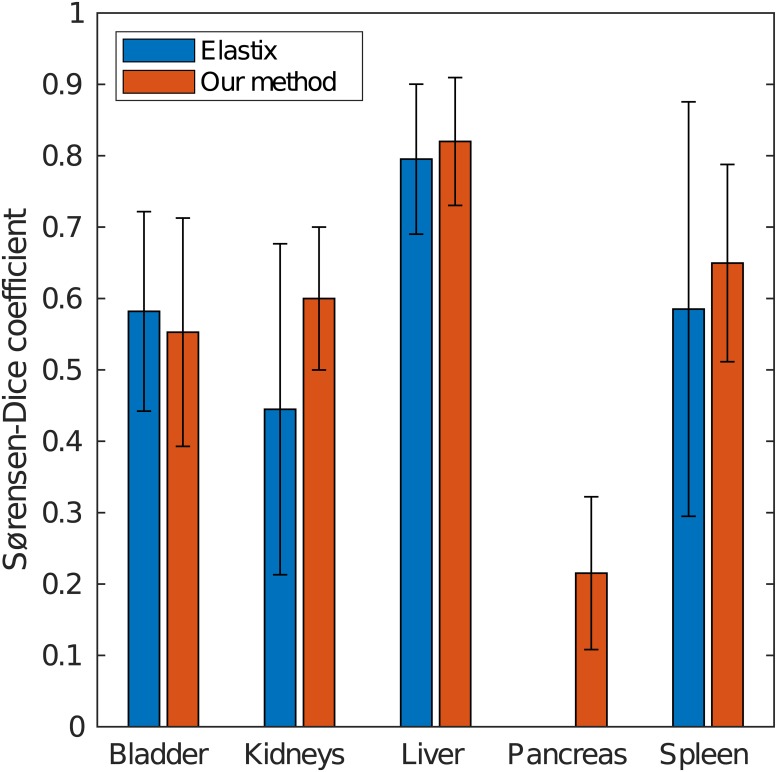
Plot of the mean and standard deviation of the Sørensen–Dice coefficient for the overlapping of segmented ROIs.

When comparing with implicit-reference groupwise registration in the third experiment, Elastix computed the groupwise registration in about 17 hours, requiring 24 GB of memory. Our method completed in about 50 minutes, requiring 1 GB of memory. The results from this experiment are reported in Table 1 and Fig 5.

**Table 1:** Mean and standard deviation of the Sørensen–Dice coefficient for the overlapping of segmented ROIs in the comparative evaluation.

ROI	Elastix	Our method	p-value^1^
Bladder	0.58(0.14)	0.55(0.16)	0.1457
Kidneys	0.44(0.23)	0.60(0.10)	0.0066
Liver	0.80(0.11)	0.82(0.09)	0.4035
Pancreas	0.00(0.00)	0.22(0.11)	0.0000
Spleen	0.59(0.29)	0.65(0.14)	0.7532

^**1**^ p–value from a paired, two–sided Wilcoxon signed–rank test, whose null hypothesis is that the difference of the outcomes from the two experiments for each subject comes from a distribution with zero median. The data for each subject is reported in S4 File.

**Fig 6:**
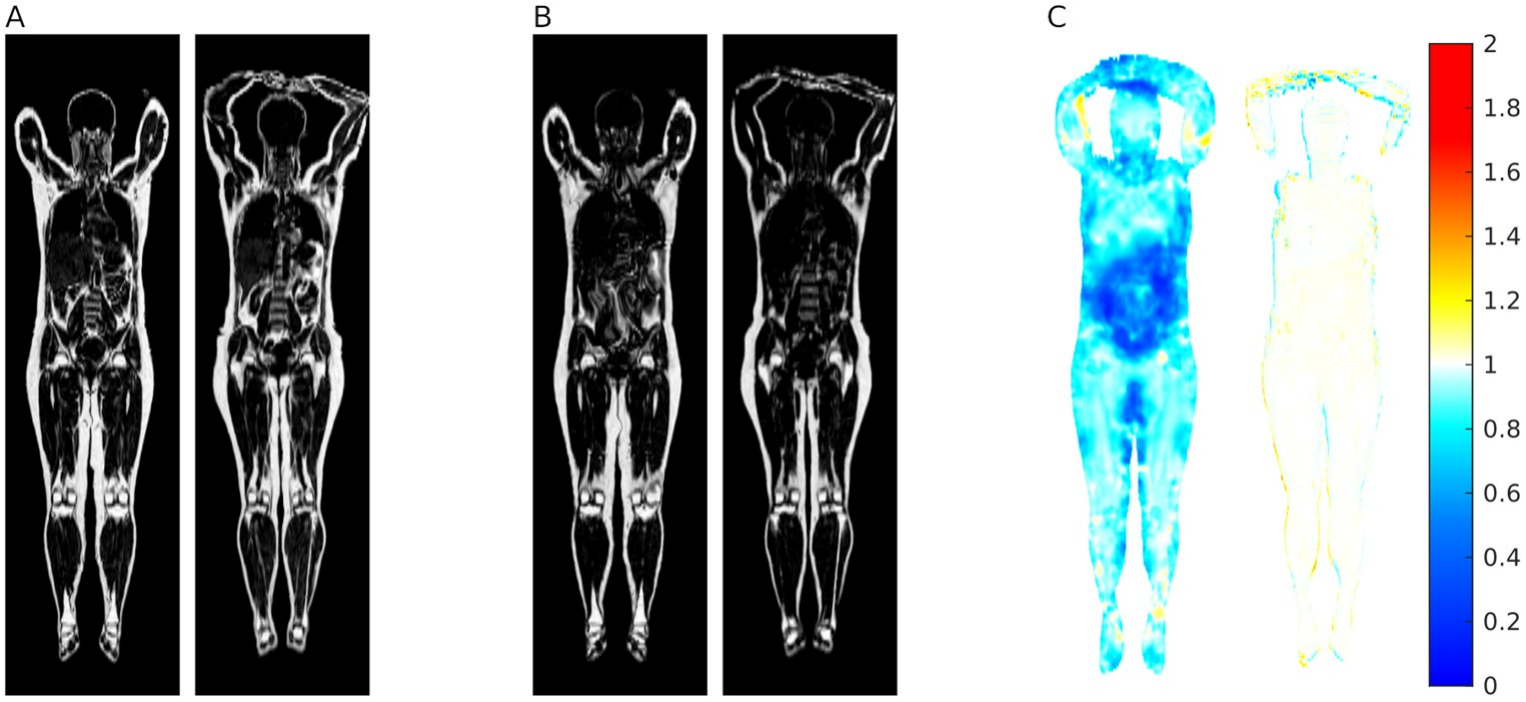
Comparison between the reference spaces generated by out method and Elastix. A: Slice of the resulting registered fat image of a subject, for Elastix (left), and our method (right). In this case, some artefacts are observable in the result from Elastix (e.g. in the subcutaneous fat), but overall the result of the registration appears to be reasonable for both methods. B: results for another subject, for Elastix (left) and our method (right). This subject is more challenging to register, and in the output from Elastix it is possible to notice visible artefacts, such as the spine being out of place, and visible distortion of the internal organs and the subcutaneous fat between the thighs. By comparison, our method does not introduce artefacts of this type. C: Average Jacobian determinant for Elastix (left) and our method (right).

From a qualitative evaluation of the results, Elastix produced acceptable registration for some subjects and poor quality results for others, with artefacts and erroneous registration, especially in the internal organs. Errors of this importance were not observed with the proposed method. Qualitative examples of registration results are presented in Fig 6.

The average Jacobian of the deformations generated by the two methods is shown in Fig 6. Since Elastix is not optimising the transforms with respect to the average Jacobian, its resulting average Jacobian is not expected to be close to one, and it is reported only for illustrative purpose.

## Discussion

The proposed method allows to generate a synthetic reference with zero pointwise average volume change. We observe a relatively high standard deviation, most voxels reach zero average volume change and the error is mainly concentrated in outliers. Imperfections in the registration process prevents to exactly reach the value of zero in a single iteration, because of several limiting factors. The significant anatomical variability in the dataset makes it hard to robustly handle all the registrations. An important source of error is the fact that the pose of the subjects is not consistent across the dataset, especially in the placement of the arms and legs, and for some subjects the extremities fall outside of the field of view of the scanner. There are also some general limitations in the quality of the alignment, for example in some regions of the thorax and abdomen, which are difficult to register accurately.

The displacement realising a given Jacobian can be generated quickly and accurately. A small increase of the average image dissimilarity is observed (Fig 3), which is attributed to the sub-voxel inaccuracy of the composition of discrete transforms. If considered significant, such variation could be cancelled with a repeated registration to refine the result, at the cost of a slightly higher average volume change, as shown in the same experiments.

The method shows a stable behaviour and while a single execution is deemed sufficient for practical applications, repeated iterations of registration and update of the reference space show a convergent behaviour toward an average pointwise volume change of zero, with most of the improvement concentrated in the first iteration (Fig 4). It is also possible to observe how the image similarity measures improve, but these changes are not deemed to be meaningful because the registration process is biased toward lower mean squared error, together with the fact that such changes in the cost function are unlikely to correspond to better registration quality [18].

This approach scales to big datasets and, for large scale studies on whole-body image volumes, it can be a practical alternative to implicit reference groupwise registration, which becomes expensive when the number and size of the images grows. In a comparison with Elastix, a deformable registration toolbox for medical image processing that implements a well established groupwise registration method, we observed better visual quality of the registered images (Fig 6). In the quantitative evaluation of ROI overlapping when mapping ground truth segmentations of five internal organs, we measured significantly higher quality (*p* < 0.01) for the kidneys and pancreas, and non significant differences for the remaining organs (Fig 5). The difference is notable in particular when observing the data for the pancreas: with Elastix the organ was completely absent from the labels in reference space, with no voxel reaching the majority vote.

## Conclusion

This work introduces a method for the generation of a reference space neutral with respect to average volume changes in the registration of multiple images, that can be inserted in a pipeline to enable fast registration of large datasets where such property would be desirable, e.g. for the purpose of statistical analysis of local tissue volume and its correlation with non-image biomarkers. Experimental results show how the method does not affect the quality of the registration process nor introduces artefacts in the anatomy of the reference space.

## Supporting information

S1 File Data for Fig 3 (female subjects). (XLS)

S2 File Data for Fig 3 (male subjects). (XLS)

S3 File Data for Fig 4. (XLS)

S4 File Data for Table 1. (XLS)
